# Burden and Depressive Symptoms Associated with Adult-Child Caregiving for Individuals with Heart Failure

**DOI:** 10.1155/2014/641817

**Published:** 2014-11-05

**Authors:** Selma Bozkurt Zincir, Murat Sunbul, Serkan Zincir, Esra Aydin Sunbul, Mustafa Oguz, Fatma Feriha Cengiz, Erdal Durmus, Tarik Kivrak, Ibrahim Sari

**Affiliations:** ^1^Psychiatry Department, Erenkoy Training and Research Hospital for Psychiatric and Neurological Disorders, 34736 Istanbul, Turkey; ^2^Department of Cardiology, Marmara University Faculty of Medicine, Fevzi Cakmak Mahallesi, Mimar Sinan Caddesi No. 41, Ustkaynarca, Pendik, 34899 Istanbul, Turkey; ^3^Department of Psychiatry, Golcuk Military Hospital, 41950 Kocaeli, Turkey

## Abstract

*Background*. The primary purpose of this study was to investigate adult-child caregiver burden in heart failure (HF) patients. Secondary purpose of the study was to identify the possible influencing factors for caregiver burden and depressive symptoms in a young adult-child caregiver group. *Methods*. A total of 138 adult-child caregivers and 138 patients with HF participated in this study. Caregivers' burden, depressive symptoms, and anxiety levels were assessed by using Zarit Caregiver Burden Scale (ZCBS), Beck Depression Inventory, and State-Trait Anxiety Inventory, respectively. *Results*. The mean ZCBS scores of the female caregivers were significantly higher than male caregivers. Approximately one-third of the adult-child caregivers had at least mild depressive symptoms. Caregivers with higher depressive symptoms had higher levels of caregiver burden. There were positive correlations between caregiving time, severity of depressive symptoms, and perceived caregiver burden. There was a negative correlation between education level of caregivers and perceived caregiver burden. Age, socioeconomic level, and marital status of patients were affecting factors for depressive symptoms in caregivers. Among caregiver characteristics, gender, marital status, and ZCBS scores seem to influence the depression in caregivers. *Conclusions*. The study findings suggest significant levels of burden and depressive symptoms even in adult-child caregivers of HF patients.

## 1. Introduction

Heart failure (HF) is a debilitating chronic disease characterized by high mortality rates, frequent hospitalizations, and an impaired quality of life. Prevalence of HF in the community is 2-3%. This ratio abruptly increases to 4–6% over the age of 65 [1]. Approximately half to two-thirds of these cases are thought to be asymptomatic. If we make a rough estimate of the studies in the world, nearly one million people in Turkey have been diagnosed with various degrees of HF [2]. The condition is a leading cause of hospitalization for elderly patients at high health care costs. Prognosis of HF is poor and two-thirds of the patients die within 5 years. Importantly, the health-related quality of life (HRQL) of these patients is poor because of the many troublesome signs and symptoms they experience [2–5]. There is growing evidence that social supportive care from family members is associated with better outcomes of HF. The extent of caregiving by the family members has increased as hospitalization period became shorter [1–4]. Family caregivers may improve the survival and enhance HRQL by providing personal and medical care, assisting with activities of daily living, monitoring symptoms, managing patient's emotions and behaviors, dealing with finances, and coordinating health services [1–5]. Thus, caregivers reduce costly hospitalizations [3, 4]. Vinson et al. found that 21% of readmissions were related to inadequate or absent social support [6]. HF patients who were single or nonmarried had a 2.1 times higher risk of readmission than married patients [5].

However, provision of support by family members may come at considerable cost to family caregivers [2]. Caregiver burden is a salient outcome of heart failure care, but it has not been well studied and tracked clinically [1, 2]. Increased perceived caregiver burden is related to higher levels of caregiver depression and anxiety [1–4]. Despite the substantial prior research available on the caregiving in other chronic diseases, the burden of family members caring for patients with chronic HF has rarely been described in detail and the factors influencing psychosocial distress and caregiver burden are poorly understood [3, 4, 7–13]. Depressive symptoms and anxiety are common psychological problems for both family caregivers and patients with chronic HF [1, 2]. Family members providing care for HF patients have indicated that it is difficult, is overwhelming, and is associated with increased anxiety, caregiver burden, and reduced quality of life [2, 4, 9].

The primary purpose of this study was to investigate adult-child caregiver burden in HF patients. Secondary purpose of the study was to identify the possible influencing factors for caregiver burden and depressive symptoms in adult-child caregiver group.

## 2. Materials and Methods

### 2.1. Study Population

This study is a cross-sectional, descriptive, and correlational study in which patients with HF and their primary caregivers completed self-report questionnaires. A nonrandom sample of 138 patients with HF and their 138 adult-child caregivers were enrolled in the study. Consecutively admitted patients, who were the care recipients, were eligible for the study if they had a documented medical diagnosis of HF and were receiving regularly scheduled clinical care at the cardiology outpatient clinic. Caregivers were eligible if they were a family caregiver, defined in this study as an adult son or daughter of a patient with HF who helps the patient at home with self-care activities and is not paid to do so, were able to speak Turkish and hear at a conversational tone, and were alert and oriented as determined by clinic staff. Thirty-four primary caregivers were excluded from the study because of having salient cognitive impairment or major comorbidities such as HF, cancer, or other terminal illnesses. The salient cognitive impairment was assessed by trained research assistants during the initial contact.

The study was approved by the local institutional review board at Marmara University in Istanbul. Data were collected from October 2012 through May 2013. The patients and their primary family caregivers were recruited from a multidisciplinary HF outpatient clinic affiliated with the university medical center. Potentially eligible patients and caregivers were invited to participate in the study by clinic staff during a regularly scheduled clinical care visit. The names of patients and their family caregivers who agreed to participate were provided to members of the research team by clinic personnel. Research team contacted the patients and caregivers to obtain signed informed consent and schedule caregiver interviews. Sociodemographic information was collected using a questionnaire consisting of six questions regarding age, gender, marital status, education level, economic level, and morbidity. All the adult-child caregivers completed the self-report questionnaires on their own. All data on the patients were retrieved from the patients' medical records at baseline by the members of research team by using a standardized data abstraction form and were entered into the database.

### 2.2. Self-Report Questionnaires

Caregivers' overall perceived caregiving burden was assessed using the Zarit Caregiver Burden Scale-Turkish version (ZCBS-TR). The ZCBS-TR has 22 items and each item is rated on a 5-point Likert scale from 0 (never) to 4 (nearly always). A total score is the sum of 22 items and ranges from 0 to 88. A cut-off total score of 17 or higher is considered to represent severe burden [14]. Although this measure was initially developed and used to assess caregiver burden for patients with dementia, there is no problem with the use of this measure for caregivers of patients with HF or coronary heart disease. ZCBS has been validated in relatives of schizophrenia patients by Ozlu et al. (2009) in Turkey [14]. Cronbach's alpha for this study was 0.83 and item total-score correlations ranged between 0.33 and 0. 75.

Depressive symptoms were assessed using the Beck Depression Inventory-II Turkish version which is a 21-item scale. Each item is rated by the participant from 0 to 3. The total score is the sum of the 21 items and can range from 0 to 63. Higher scores indicate higher levels of depressive symptoms. Caregivers who received a score of 14 or above were considered to have at least mild depressive symptoms. Turkish version (BDI-TR) has been validated by Hisli (1989) in Turkey [15]. Cronbach's alpha for this study is 0.80.

Anxiety symptoms were assessed using the State-Trait Anxiety Inventory-Turkish version (STAI-TR). STAI is a 40-item scale involving state and trait anxiety subscales, each subscale has 20 items, and some items are reverse-scored. The total scores of both subscales range from 20 to 80. There was no established cut-off score for STAI-TR scale. Higher scores indicate higher levels of anxiety symptoms. STAI was developed in order to determine the levels of state and trait anxiety separately by Spielberg and his colleague in 1970 which stemmed from the Spielberger two-factor theory of anxiety. The State Anxiety Inventory (STAI-1) requires an individual to describe how he feels himself at a specific time and specific circumstances and taking into account his feelings on the situation in which to answer. The Trait Anxiety Scale (STAI-2) requires the individual to describe generally how he feels himself. Turkish version of State-Trait Anxiety Inventory has been validated by Oner (1978) in Turkish population [16]. Cronbach's alpha for this study was 0.83 and 0.92 for state and trait subscales, respectively.

We also collected caregivers' age, gender, marital status, education and socioeconomic level, relationship to the patient, and some of the patient's demographic and clinical characteristics (NYHA class, comorbid diseases, and left ventricular ejection fraction) using a structured questionnaire. When the ejection fraction was indicated as a range, the mean value was used.

### 2.3. Statistical Analyses

Statistical analyses were performed using SPSS 15.0 version of statistical package for Windows. Descriptive statistics including frequency, percentile, mean, and standard deviation were used to describe variables used in this study. Continuous data were expressed as mean ± standard deviation while categorical data were presented as percentage. Chi-square test was used for comparison of categorical variables while Student's *t*-test or Mann-Whitney *U* test was used to compare parametric and nonparametric continuous variables, respectively. Normal distribution was assessed by Kolmogorov Smirnov test. Correlation analysis was performed by Pearson or Spearman's correlation test. Linear regression analyses were performed to determine the possible affecting factors for caregiver burden and depressive symptoms of caregivers. *P* value under 0.05 was considered statistically significant for all data analysis.

## 3. Results

Baseline characteristics and clinical data of HF patients and adult-child caregivers were shown in Table 1. A total of 138 HF patients and their adult-child caregivers participated in this study. Patients with HF were relatively stable outpatients who had no acute admission episodes within the previous six months. As shown in Table 1, their mean age was 66.36 ± 12.71 and 45.7% of patients (*n* = 63) were male. They had either preserved or nonpreserved left ventricular function with a mean ejection fraction of 36.6%. 31.4% of the patients had an ischemic etiology underlying their HF. Approximately two-thirds of the patients were in NYHA classes III and IV. Majority of the patients had at least one to three comorbidities. The most common comorbidities were hypertension and diabetes mellitus. The mean age of adult-child caregivers was 37.13 ± 11.57 years with a range between 20 and 55 years. Caregivers were an average of 29 years younger than the patients. The majority of adult-child caregivers were female (65.2%) and had only primary education (≤8 year) and about one-third had reported low socioeconomic status. The mean caregiver time was 44.36 ± 34.79 months (min: 10 months, max: 120 months).

Comparison of the psychiatric status of caregivers according to gender was shown in Table 2. All adult-child caregivers had severe caregiving burden (ZCBS ≥ 17) and female caregivers had significantly higher ZCBS scores compared to male caregivers. Approximately one-third (34.8%) of the adult-child caregivers had at least mild depressive symptoms (BDI ≥ 14). There was no significant difference in terms of severity of depressive symptoms between male and female adult-child caregivers. On the other hand, female adult-child caregivers had significantly higher anxiety scores on both STAI-1 and STAI-2 subscales. Adult-child caregivers with primary educational level (≤8 years) have significantly higher levels of caregiver burden compared to adult-child caregivers with educational level >8 years (40.37 ± 7.46 versus 34.09 ± 6.86, *P* = 0.001).

Correlation analysis of caregiver burden with caregiving time period and psychiatric scale scores was shown in Table 3. There were positive correlations between caregivers burden and caregiving time period (*r* = 0.327, *P* < 0.001). The mean caregiving time for longer than >36 months was 40.17 ± 8.39 and the mean caregiving time for lower than <36 months was 34.56 ± 6.07. There were significantly higher levels of caregiver burden in adult-child caregivers of patients with longer (>36 months) caregiving time period (*P* = 0.001, *t* = 4.49). There was also positive correlation between depressive scores of caregivers and caregiver burden (*r* = 0.410, *P* < 0.001). We detected negative correlation between educational levels of caregivers and perceived caregiver burden (*r* = −0.31, *P* < 0.001).

Linear regression analysis revealed that age, socioeconomic level, and marital status of HF patients were affecting factors for depressive symptoms in adult-child caregivers. Neither severity of HF nor other clinical characteristics of HF patients were related to depressive symptoms in adult-child caregivers. Among adult-child caregiver characteristics, gender, marital status, and ZCBS scores seem to influence the depression in caregivers (Table 4).

## 4. Discussion

In this study, we focused on caregiver burden and depressive symptoms among the adult-child caregivers of stable HF outpatients who had not been hospitalized within the last six months of baseline. Our cross-sectional study reveals some important findings about younger adult-child caregivers burden for a relatively younger heart failure patient population. We found that more than one-third of adult-child caregivers (34.8%) experienced at least mild depressive symptoms. Additionally, female caregivers had significantly higher scores on both STAI-1 and STAI-2 subscales as well as ZCBS scale. Severity of depressive symptoms was higher in female caregivers but this difference did not reach significance. In a previous study, Saunders found that higher caregiver burden is significantly associated with certain elements within the caregiving environment and certain caregiver characteristics (caregivers with high risk profiles), such as more patient comorbidities, lack of respite caregiver, increasing caregiver depressive symptoms, more caregiver hours, older age and/or multiple caregiver health problems, and caring for other relatives [17]. Similar to the findings by Saunders our study suggests that caregiver burden shared a strong relationship with depressive symptoms.

The prevalence of depressive symptoms among caregivers in this study was consistent with previous reports of caregivers of patients of HF [2, 4, 8, 9, 18]. Considering the relatively stable condition of patients engaged in self-management in the home settings in the present study, the prevalence of depressive symptoms in primary family caregivers was significant. According to the findings of previous studies on family caregivers of patients with HF, primary caregivers who had depressive symptoms at baseline were more likely to report their life was changed negatively and their mental quality of life was poor at followup [3, 4, 19–22].

Consistent with previous studies, we also detected that family caregivers who had longer caregiving time, higher levels of anxiety, and depressive symptoms had also more severe caregiver burden. In our study, caregivers with lower educational level (≤8 years) reported significantly higher levels of caregiver burden. In Chung's study, it has been shown that depressive symptoms of caregivers were predicted by their own individual characteristics and severe perceived burden [2]. Similarly, in our study, we found that depressive symptoms of caregivers were related with their gender (being female), marital status (being single or nonmarried), and severe perceived burden. In the present study, the amount of time spent in caregiving seems to be affecting depressive symptoms. Based on this we can say that caregivers' depressive symptoms may be related to their individual characteristics rather than severity of patient's disease or functional impairment. This result was consistent with Chung's research findings [2, 5]. Among the possible causes of caregiver burden there may be many factors (e.g., extent of coping strategies, mastery in caregiving tasks) which cannot be evaluated in this study.

Another noteworthy finding in this study was that, in our study, adult-child caregivers burden was investigated. Because the vast majority of the primary caregivers (>90%) of our scheduled care-visit patients with heart failure were their adult children, we included only the adult-child caregivers in our study in order to homogenize the sample. In addition, the majority of the spouses of married HF patients, due to their own illness or personal inadequacies, were not in the position of primary caregiver. So, adult-child caregivers who constitute our study group did not have much chance for respite caregiving. Saunders and some other researchers reported that family support exerts a positive influence on how HF caregivers perceive their situation [23–25]. Respite caregiving lessened family burden (spouse and adult-child caregivers) and is a likely alternative approach to care [17]. This means that caregivers prefer and desire the respite achieved with the assistance of another family caregiver (adult-child or spouse) [23, 26].

In the present study, adult-child caregivers' depressive symptoms were not predicted by severity of HF disease (NYHA class) and other clinical characteristics of HF patients. On the other hand, age, socioeconomic level (having lower income), and marital status (being single) of the HF patients seem to be possible influencing factors for depressive symptoms of adult-child caregivers. In fact, caregiver burden which often reflects the level of dependency of the care recipient was different between those that had few depressive symptoms and those with mild and great symptoms indicating that it is related to severity of disease. HF class may not have distinguished the caregiver depression and HF class may not be the best indicator of the amount of dependency of the care recipient in the home environment [2, 19–21].

In the present study, all of the adult-child caregivers experienced severe levels of caregiver burden which is consistent with findings of previous research indicating that informal caregiving in family members of patients with a variety of chronic diseases also brought new roles and responsibilities leading to worse psychological and physical health for the caregiver [4, 13, 27]. According to the findings of this study, adult-child caregivers' burden was related to gender (being female), socioeconomic level (lower income), educational level (≤8 year), severity of depressive symptoms, and caregiving time.

According to the findings of our study, it is noteworthy that family caregivers are usually expected to undertake care providing despite their own physical disability or psychological distress or burden. Family caregivers for the most part do not care about their own physical and mental health needs because they focus on HF patients. Eventually, they often become overwhelmed and neglect their own health, further increasing the potential for burnout. Health care professionals should be aware of the fact that family caregivers who perceive medium and high levels of caregiver burden are at risk for feeling ill themselves [4, 27].

### 4.1. Study Limitations

There are some limitations of the present study. Firstly, this study is limited by its cross-sectional study design in terms of explanation of causal relationship between level of psychosocial functionality and the caregiver role. Because our study group is composed of relatively young adult-child caregivers, research findings may only be generalized to adult-child caregivers with caution. Other variables which were not measured in the present study could have contributed to caregiver burden among family members of patients with HF. Secondly, the sample size was relatively small, which can partially be attributed to the larger number of patients with chronic HF screened who did not live with a family member. Thirdly, our hospital is a regional public university hospital that appeals to a wide public. So the majority of patients with HF when admitted to us might be more extreme and their caregivers were the ones volunteering to participate. Thus, the results cannot be generalized for the entire population of caregivers. A fourth limitation of this study was that the gender distribution in our study was unequal but reflects the reality that women provide informal care more often than men [3, 28]. Despite these study limitations, this study is one of the few studies published to date on family caregivers of HF patients.

## 5. Conclusions

The study findings suggest that health care providers should assess depressive symptoms or burden related to caregiving role regularly in order to prevent negative health outcomes for both HF patients and their adult-child caregivers. The depressive symptoms that occur among caregivers may have physiological and psychological deleterious effects, especially if primary caregivers were alone and fully responsible for the care. The findings of this study suggest that it might be important to identify and screen depressive symptoms of caregivers before they reach “burnout” in order to provide optimal quality of care to patients with HF and to prevent poor health outcomes of family members. Further large scale researches are needed to examine whether improvement of depressive symptoms in caregivers will have a positive impact on the course of caregivers as well as patients.

## Figures and Tables

**Table 1:** Clinical characteristics and demographic data of study population.

Demographic data	Patients (*n* = 138)	Caregivers (*n* = 138)
Age (years)	66.4 ± 12.7	37.1 ± 11.6
Gender, male (%)	63 (45.7)	48 (34.8)
Education status (primary school), *n* (%)	81 (58.7)	72 (52.1)
Marital status (married), *n* (%)	90 (65.2)	99 (71.7)
Low socioeconomic status, *n* (%)	69 (50)	48 (34.8)

Clinical characteristics of HF patients	Patients (*n* = 138)	

Hypertension, *n* (%)	75 (54.3)	
Diabetes mellitus, *n* (%)	63 (45.7)	
Coronary artery disease, *n* (%)	33 (31.4)	
Smoking, *n* (%)		
No	36 (26.1)	
Yes	102 (73.9)	
Ejection fraction (%)	36.6 ± 11.9	
Serum creatinine levels (mg/dl)	1.34 ± 0.82	
Pro-BNP levels (pg/mL)	6940.1 ± 9659.3	
NYHA functional classification, *n* (%)		
Class 1	0	
Class 2	51 (37)	
Class 3	66 (47.8)	
Class 4	21 (15.2)	

Data are presented as mean ± standard deviation or number of study samples.

HF: heart failure; NYHA: The New York Heart Association; Pro-BNP: pro-brain natriuretic peptide.

**Table 2:** Comparison of the psychiatric scale scores of caregivers according to gender.

Psychiatric scale scores	All caregivers (*n* = 138)	Male (*n* = 48)	Female (*n* = 90)	*P*
BDI	12.2 ± 8.6	11.4 ± 7.3	13.8 ± 10.5	0.111
STAI-1	41.5 ± 3.8	40.6 ± 3.6	43.3 ± 3.6	0.001
STAI-2	47.2 ± 6.8	45.6 ± 6.5	50.3 ± 6.2	0.001
ZCBS	37.4 ± 7.8	34.7 ± 7.9	38.8 ± 7.4	0.003

Data are presented as mean ± standard deviation. BDI: Beck Depression Inventory; STAI-1: State Subscale of State-Trait Anxiety Inventory; STAI-2: Trait Subscale of State-Trait Anxiety Inventory; ZCBS: Zarit Caregiver Burden Scale.

**Table 3:** Correlation analysis of caregiver burden with sociodemographic data and psychiatric clinical scales.

	*r*	*P*
Caregiving time period	0.327	**<0.001**
Beck Depression Inventory	0.410	**<0.001**
State Subscale of State-Trait Anxiety Inventory	0.130	0.120
Trait Subscale of State-Trait Anxiety Inventory	0.050	0.510
Caregivers' education level	−0.310	**<0.001**

**Table 4:** Linear regression analysis for depressive symptoms of caregivers.

Clinical characteristics	*β*	*t*	*P*	95% confidence interval	
				Minimum	Maximum
Age of patient	0.281	3.134	**0.002**	0.071	0.316
Age of caregiver	−0.034	−0.294	0.769	−0.244	0.181
Gender of patient	0.148	1.562	0.121	−0.698	5.913
Gender of caregiver	−0.227	−2.458	**0.015**	−7.430	−0.799
Educational status of patient	0.026	0.261	0.795	−2.357	3.072
Educational status of caregiver	−0.084	−0.957	0.341	−2.703	0.942
Socioeconomic status of patient	0.281	4.160	**<0.001**	3.490	9.832
Socioeconomic status of caregiver	−0.136	−1.465	0.146	−5.989	0.895
Marital status of patient	0.206	2.315	**0.022**	3.486	0.272
Marital status of caregiver	0.270	2.640	**0.009**	1.360	9.523
ZCBS	0.403	4.834	**<0.001**	0.262	0.626
STAI-1	0.024	0.282	0.778	−0.331	0.441
STAI-2	0.070	0.855	0.394	−0.118	0.297
CT	0.150	1.763	0.080	−0.004	0.078

ZCBS: Zarit Caregiver Burden Scale; STAI-1: State Subscale of State-Trait Anxiety Inventory; STAI-2: Trait Subscale of State-Trait Anxiety Inventory; CT: caregiving time.
